# Hospital Finance

**Published:** 1922-05

**Authors:** 


### HOSPITAL FINANCE.

### THE GREAT SUCCESS OF THE OXFORD SCHEME.

THE remarkable success of the Radcliffe Infirmary, Oxford, contribution scheme was explained by the Rev. G. B. Cronshaw, the treasurer, to the recent half-yearly meeting of the secretaries on whose local efforts the district contributions depend. Their attendance at the hospital on these occasions not only shows to them the work accomplished by their aid, but brings them into contact with each other and with the hospital. It is hoped that the contributors will be represented on the hospital's committee, so that the hospital's needs and difficulties can be known as intimately to the contributors' delegates as to the committee itself, and that the hospital can play its proper part in the country areas. The scheme, which enabled the hospital to pay its way last year, raised £17,520, of which £11,000 was subscribed by towns and villages, and £6,000 by firms, work and colleges. The interest stimulated was so gr at that a further £8,000 was received through donations. The movement, in short, is now relied on for £25,000 a year: an astonishing result in a mainly agricultural area. This sum is two-thirds of the total (£38,000) required to main'ain the hospital. Dr. W. Collier, shortly after the meeting just referred to, explained in "The Times" that the success of the scheme was largely due to the medical men and others, who have explained to organised local meetings the enormous amount of wo k which a county hospital carries on by the gratui ous work of the medical staff. From this he infers that the scheme would come to disaster if the medical men, as some have suggested, received a percentage of the contributions. Such a plan, he adds, would not seem fair to the general practitioners. The hospital's medical staff, here as elsewhere, recognised an appointment to be in itself a considerable reward; and it is probably true to say, as the voting at the conference on March 22 suggested, that the staffs of the large hospitals, in particular, and of many others also are still opposed to payment for their services. To have devised a scheme of patients' contributions which leaves the old medical tradition untouched, shows the elasticity of the voluntary system. It would be impossible under any form of public control; and for an agricultural community to vie with an industrial centre in the amount raised from weekly contributions is a no less remarkable feat. It shows that the voluntary system can support itself under any conditions, for even the difficult problem of London promises to be solved in time by similar means.

### LIVERPOOL EMPLOYERS AND THE HOSPITALS.

MR. C. KENNETH, honorary secretary of the Liverpool Hospital Saturday Fund, has made some useful suggestions for improving the collections in Liverpool, which, like those in Manchester, are far behind the totals raised even in smaller cities. Why is this? Mr. Kenneth, who must know, declares that many Liverpool employers refuse to allow weekly collections even when their workpeople are ready to contribute. He adds the hardly less astonishing fact that many employers, who are on the committees of the hospitals, have failed to encourage contributions from their staffs. This is an almost incredible condition of affairs, and a very damaging indictment against the state of public spirit in Liverpool. A campaign to drive these facts home would do more, we feel, to remedy them than Mr. Kenneth's proposal that only firms now making a weekly contribution should be eligible to tender for hospital contracts. The latter would excite antagonism, which never benefits the cause of charity. The former would rally all decent people to the hospitals' side, and throw the abstainers on their defence. Obviously, a beginning should be made with those employers who are already members of hospital committees. The attitude of these men seems wholly indefensible.

### A LEAD FROM THE MIDLAND RAILWAY.

MR. JAMES BIRKETT, honorary treasurer of the Liverpool Hospital for Women, lately alluded to the proposal of the Midland Railway Company to make a weekly contribution in addition to that of its staff to the voluntary hospitals, in order to preserve the voluntary system, and to save the shareholders from the greater expense of public hospitals. Mr. Birkett pointed out that the arguments in favour of the Midland scheme applied to all commercial concerns, and he hoped that business firms all over the country would follow suit. If they did it would be a credit to Liverpool, for not only was Mr. Charles Booth, till he announced his resignation, chairman of the Midland Railway, but he was also chairman of the Liverpool Council of Voluntary Aid. Mr. Birkett's proposal was therefore that every firm in Liverpool should contribute a sum equal to a penny in the pound of its rateable value. Such a sum, the product of 1d. rate, was £21,000. The estimated annual deficiency of the Liverpool hospitals was £40,000, so that obviously municipal control would mean a 2d. rate to begin with. Despite all difficulties sectional subscriptions have spread from the industrial north to London, and there seems no reason why business firms should not fall into line.

### COTTAGE HOSPITALS AND CONTRIBUTION SCHEMES.

NOW that the Braintree and Bocking Cottage Hospital has moved to its new quarters at the former place, which the generosity of Mr. W. J. Courtauld has made possible, the problem of maintaining the larger institution is pressing. To solve it a scheme has been devised whereby employees of firms which subscribe to the hospital shall have priority of treatment over the general public. If this is the substance of the plan, it is open to criticism. It is the tradition of the voluntary hospital that priority of admission shall be based solely on the urgency of the case. Any deviation from this tradition is open to grave objection. In similar schemes sometimes no privilege is given to contributing firms, but when a privilege is granted it usually takes the form of reducing or abolishing the maintenance charges now generally made upon all patients who can afford to contribute. The argument "The hospital will look after you when you are ill, if you will remember it when in health" is reasonable, but, so far as we are aware, it has never been interpreted to mean priority of treatment. Mr. Courtauld, the president, is a veteran hospital worker, unlikely to approve of any questionable plan.

### HOSPITAL LETTERS AND OUT-PATIENTS' CHARGES.

A CONFERENCE of representatives of various mutual and working men's societies was lately held in East London to consider complaints made by out-patients attending with letters against the charges made for treatment. It was urged that, as these letters were given only to those unable to pay for medical advice, it was a hardship to exact a payment from them. It was resolved to ask the various hospitals concerned to discontinue the practice. Though the letter system is old, it has never been popular, and, indeed, often causes considerable suffering to patients in the long search which is often required before any letter can be obtained. The system is defended because it is supposed to attract subscribers, who enjoy the petty patronage conferred by the possession of a letter, as much as the needy suffer pain seeking it. Nor is there much logic in presenting a subscriber with a letter securing admission to a patient if the patient has to contribute for his treatment. We do not say that such a charge in present circumstances in unreasonable. We say, rather, that a fresh argument has been produced by the charge against the letter system; and we do not believe that in the long run any hospital need suffer which abolishes it.

### INSURANCE AGAINST OPERATIONS.

AN interesting suggestion, which, however, needs further explanation, has been made by a correspondent in the "Birmingham Daily Mail," who says: "I have subscribed weekly and otherwise (to hospitals) for over 30 years, and was recently ordered an immediate operation, so had to enter the hospital as a private patient. The cost altogether was nearly £50, which took a large part of my life's savings." He therefore proposes that working-men subscribers, in addition to their weekly pence, should pay, say, 15s. a year, if the hospital's authorities. agreed that this should entitle to a free operation, if required, all wage-earners who were weekly subscribers. It is not clear what he means by adding that the scheme is applied to some hospitals in London. Contribution schemes no doubt carry different privileges in different places, but how came it that a weekly contributor of over 30 years' standing had to enter hospital as a private patient?

### GRANTS OR RATE ABATEMENTS AT CARDIFF.

AT the time of writing, it seems probable that Sir William Diamond's efforts to gain a more generous grant from the Cardiff Corporation to King Edward VII. Hospital will be successful. He has been pressing the hospital's case for some time with equal tact and persistence, and the Cardiff Finance Committee has agreed to recommend to the City Council that the grant shall be increased from £500 to £1,500 a year. The latter figure is the sum suggested by Sir William. It is noteworthy that his suggestions for relief in the scale charged to the hospital for rates, water and electricity received a different reception from the Finance Committee. The City Treasurer brought forward figures to show that the hospital was modestly rated, and though opinion was divided, the proposal to increase the grant rather than to reduce the rates carried the day. Rules and abatements in special cases certainly complicate official dealings, and a low assessment for rates, together with a reasonably generous grant, is probably the most statesmanlike system. Sir William Diamond's advocacy certainly deserves the reward which is now apparently within his reach.

### ENCOURAGEMENT FROM GLASGOW.

TO take the condition of a Glasgow hospital as an index of the voluntary system is a severe test, but the report of the Glasgow Royal Infirmary for 1921 is not discouraging. While the ordinary revenue shows an increase in annual subscriptions and employees' contributions, with a decrease in donations, the net deficiency of £36,357 was more than met by extraordinary revenue, chiefly special donations. This enabled a sum of £1,060 to be placed to capital reserves. Of course, extraordinary revenue fluctuates and can be no permanent solution of the financial problem, but the hospital does such an amount of work, notably in the vast numbers who attend the out-patient department, that there should be a nucleus of public support in the city ready for development, since it is doubtful how far the families from which the out-patients are drawn are co-extensive with the employees who weekly contribute. The annual subscriptions so badly wanted should include an accession of those who subscribe a small sum each week.

### INCOME TAX AND SPECIAL DONATIONS.

AN interesting passage is contained in the annual report of the Queen's Hospital, Birmingham. It affirms that the Inland Revenue authorities will "raise no objection to special contributions being regarded, to an extent considered reasonable in all the circumstances of the particular case, as deferred annual subscriptions, and to their being allowed like ordinary annual subscriptions as a trading expense for the purpose of income tax."

